# History of kidney stones and risk of chronic kidney disease: a meta-analysis

**DOI:** 10.7717/peerj.2907

**Published:** 2017-01-24

**Authors:** Weifeng Shang, Lixi Li, Yali Ren, Qiangqiang Ge, Ming Ku, Shuwang Ge, Gang Xu

**Affiliations:** 1 Department of Nephrology, Tongji Hospital affiliated with Tongji Medical College, Huazhong University of Science and Technology, Wuhan, China; 2 Department of Medical Affaires, Liyuan Hospital affiliated to Tongji Medical College, Huazhong University of Science and Technologyy, Wuhan, China; 3 Department of Urology, Tongji Hospital affiliated with Tongji Medical College, Huazhong University of Science and Technology, Wuhan, China

**Keywords:** Meta-analysis, Nephrolithiasis, Chronic kidney disease, Kidney stones

## Abstract

**Background:**

Although the relationship between a history of kidney stones and chronic kidney disease (CKD) has been explored in many studies, it is still far from being well understood. Thus, we conducted a meta-analysis of studies comparing rates of CKD in patients with a history of kidney stones.

**Methods:**

PubMed, EMBASE, and the reference lists of relevant articles were searched to identify observational studies related to the topic. A random-effects model was used to combine the study-specific risk estimates. We explored the potential heterogeneity by subgroup analyses and meta-regression analyses.

**Results:**

Seven studies were included in this meta-analysis. Pooled results suggested that a history of kidney stones was associated with an increased adjusted risk estimate for CKD [risk ratio (RR), 1.47 95% confidence interval (CI) [1.23–1.76])], with significant heterogeneity among these studies (*I*^2^ = 93.6%, *P* < 0.001). The observed positive association was observed in most of the subgroup analyses, whereas the association was not significant among studies from Asian countries, the mean age ≥50 years and male patients.

**Conclusion:**

A history of kidney stones is associated with increased risk of CKD. Future investigations are encouraged to reveal the underlying mechanisms in the connection between kidney stones and CKD, which may point the way to more effective preventive and therapeutic measures.

## Introduction

Kidney stones, solid concretions or crystal aggregations formed in the kidneys from dietary minerals in the urine, are common and responsible for significant morbidity. Racial differences are seen in kidney stone disease, primarily occurring in whites, followed by Hispanics, blacks, and Asians (Romero, Akpinar & Assimos, 2010). Kidney stone prevalence also varies by age and sex. In the United States, the lifetime prevalence of kidney stones is 12% among men and 7% among women (Pearle, 2001; Pearle, Calhoun & Curhan, 2005). Over the past decade, kidney stones have become increasingly recognized as heralds of certain systemic disorders, including subclinical atherosclerosis, hypertension, diabetes, metabolic syndrome, and cardiovascular disease (Ferraro et al., 2013). All of the above conditions are known risk factors for chronic kidney disease (CKD). In this context, a number of epidemiologic studies assessed the relationship between a history of kidney stones and CKD. Although a previous review performed by Keddis & Rule (2013) has summarized the association between kidney stone history and an increased risk of CKD, the review has not provided an overall estimation of the effect of kidney stones on CKD. Data from two cross-sectional studies have shown that kidney stones were associated with CKD (Ingsathit et al., 2010; Shoag et al., 2014). However, it is difficult to establish the detrimental effects of kidney stones on CKD. Across two case-control studies, patients with kidney stone history had about a two-fold higher risk for CKD (Keller, Chen & Lin, 2012; Vupputuri et al., 2004). In addition, several cohort studies have also showed increased risk of CKD in patients with kidney stones (Alexander et al., 2012; Ando et al., 2015; Hippisley-Cox & Coupland, 2010; Rule et al., 2009), nevertheless, not all studies have shown a similar association (Kummer et al., 2015). Therefore, a meta-analysis of case-control and cohort studies was performed to qualify and quantify the risk, which may highlight the importance of providing additional intervention methods in this area.

### Methods

This study was conducted according to the Preferred Reporting Items for Systematic Reviews and Meta-Analyses (PRISMA) statement checklist (Supplemental Information 1) (Stroup et al., 2000).

### Search strategy

PubMed and EMBASE were searched for observational studies to November 4, 2015, using the terms “renal stones” or “renal stone” or  “kidney stones” or “kidney stone” or “nephrolithiasis” and “chronic kidney disease” or “kidney failure” or “renal disease” or “kidney disease” or “renal insufficiency” or “renal failure” or “kidney failure” and “risk” or “incidence” or “epidemiology.” With these parameters, two investigators (WS and LL) independently filtered out all the eligible articles and hand-searched references of retrieved papers for additional available studies. Conflicting results achieved consensus by discussion.

### Inclusion criteria

The inclusion criteria were: (1) case-control or cohort studies involving participants 18 years or older; (2) provided the multivariate-adjusted odds ratio (OR), risk ratio (RR), hazard ratio (HR), or standardized incidence ratio (SIR) with 95% confidence interval (CI), or sufficient information to calculate these; and (3) a comparison group made up of participants without kidney stones history was used.

### Exclusion criteria

The exclusion criteria were: non-human studies, reviews, comments, editorials, case reports, and cross-sectional studies. If a cohort study was reported in more than one publication, we chose the latest article.

### Data extraction and quality evaluation

The following data were extracted from the included studies: first author’s name, year of publication, study design, country, sample size, mean age, mean follow-up period, method of kidney stones and CKD diagnosis, participants with baseline CKD excluded (yes or no), and adjustment factors. When needed, we contacted the original author for clarification. The quality of each study was independently evaluated by two investigators (WS and YR) using the Newcastle-Ottawa Scale (NOS) (Wells et al., 2000). Articles scoring 0–3, 4–6, and 7–9 were defined as poor, fair and good quality, respectively. Discrepancies between investigators were solved by consensus.

### Statistical analysis

The studies included in the meta-analysis reported different effect measures (odds ratio or hazard ratio). The HR in each primary study was directly considered as RR (Spruance et al., 2004). The OR was transformed into RR by the formula RR = OR∕[(1 − P_0_) + (P_0_ × OR)] (where P_0_ is the incidence of the outcome of interest of the nonexposed group) (Zhang & Yu, 1998). The combined RR and the corresponding 95% CI were obtained using the random-effects method of DerSimonian & Laird (1986). The heterogeneity of RR across the studies was assessed with Chi-squared based Q-statistic test (*P* < 0.10) and quantified using I^2^ index (Higgins et al., 2003), where 25%, 50% and 75% indicate low, moderate and high heterogeneity, respectively. Sources of heterogeneity were explored by subgroup analyses and univariable random-effects meta-regression. Stratified analyses were conducted based on study design (case-control or cohort), region (Asian or non-Asian), sample size (<50,000 or ≥ 50,000), participant’s average age (<50 or ≥ 50 years), mean follow-up (<7.5 or ≥ 7.5 years), and gender (men or women). Sensitivity analyses were conducted to assess the robustness of results by sequential omission of individual studies (Copas & Shi, 2000). All analyses were performed in Stata 10.0 (College Station, TX, USA). A 2-tailed *P* value < 0.05 was considered significant.

**Figure 1:**
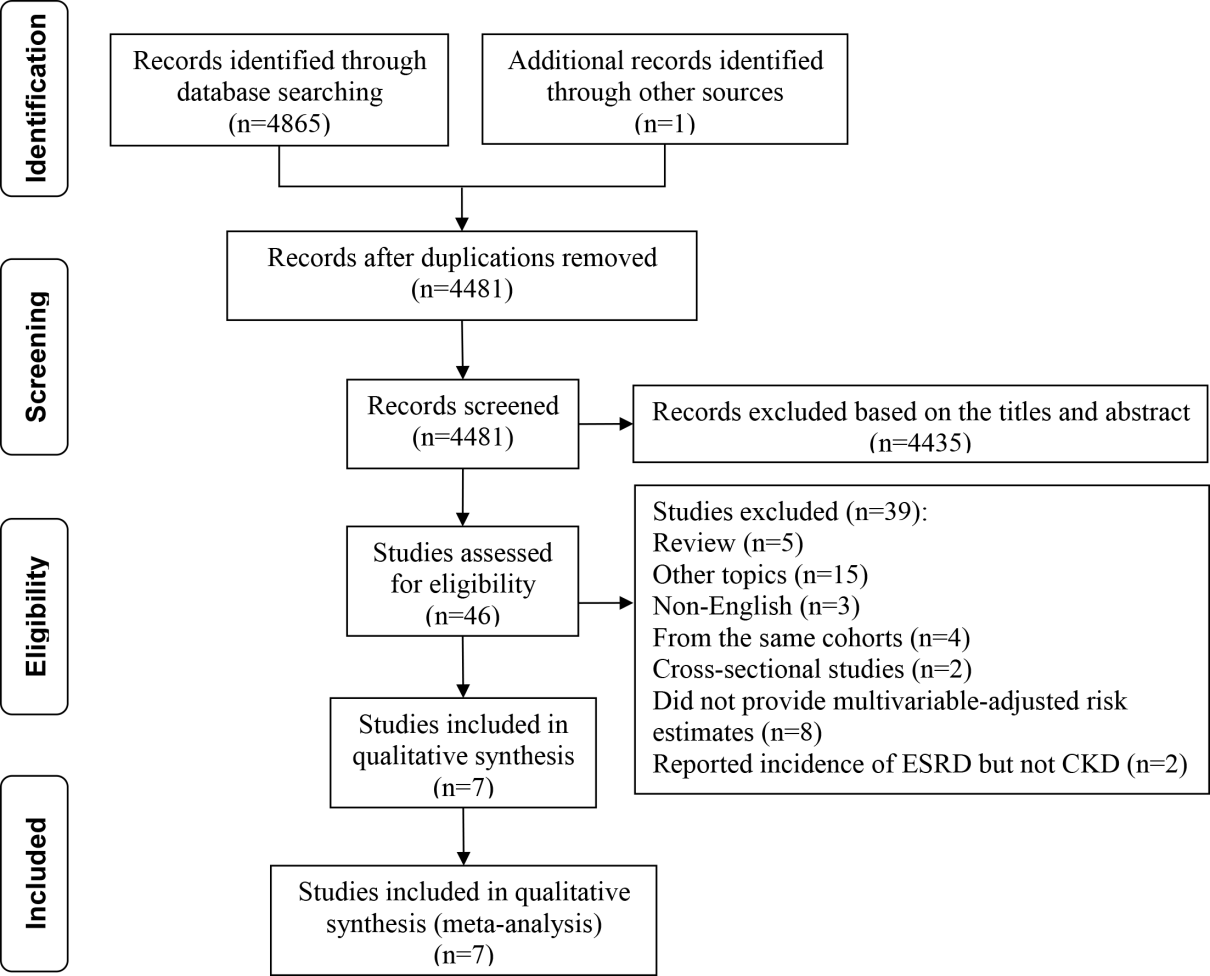
Flow chart of study selection.

## Results

### Study selection, characteristics, and quality

As shown in Fig. 1, our literature search returned 4,866 results for relevant articles, and the full text was retrieved for 46 articles, from which we identified seven observational studies for inclusion in the meta-analysis (Keller, Chen & Lin, 2012; Vupputuri et al., 2004; Alexander et al., 2012; Ando et al., 2015; Hippisley-Cox & Coupland, 2010; Rule et al., 2009; Kummer et al., 2015). One study was published in abstract form, and all other studies were in full text form. The main characteristics of the included studies are presented in Table 1. The primary analysis included data for 2,810,233 participants derived from seven observational studies. According to the NOS, six studies were of good quality and one of fair quality (Table S1).

### Risk of kidney stones on CKD events

**Figure 2:**
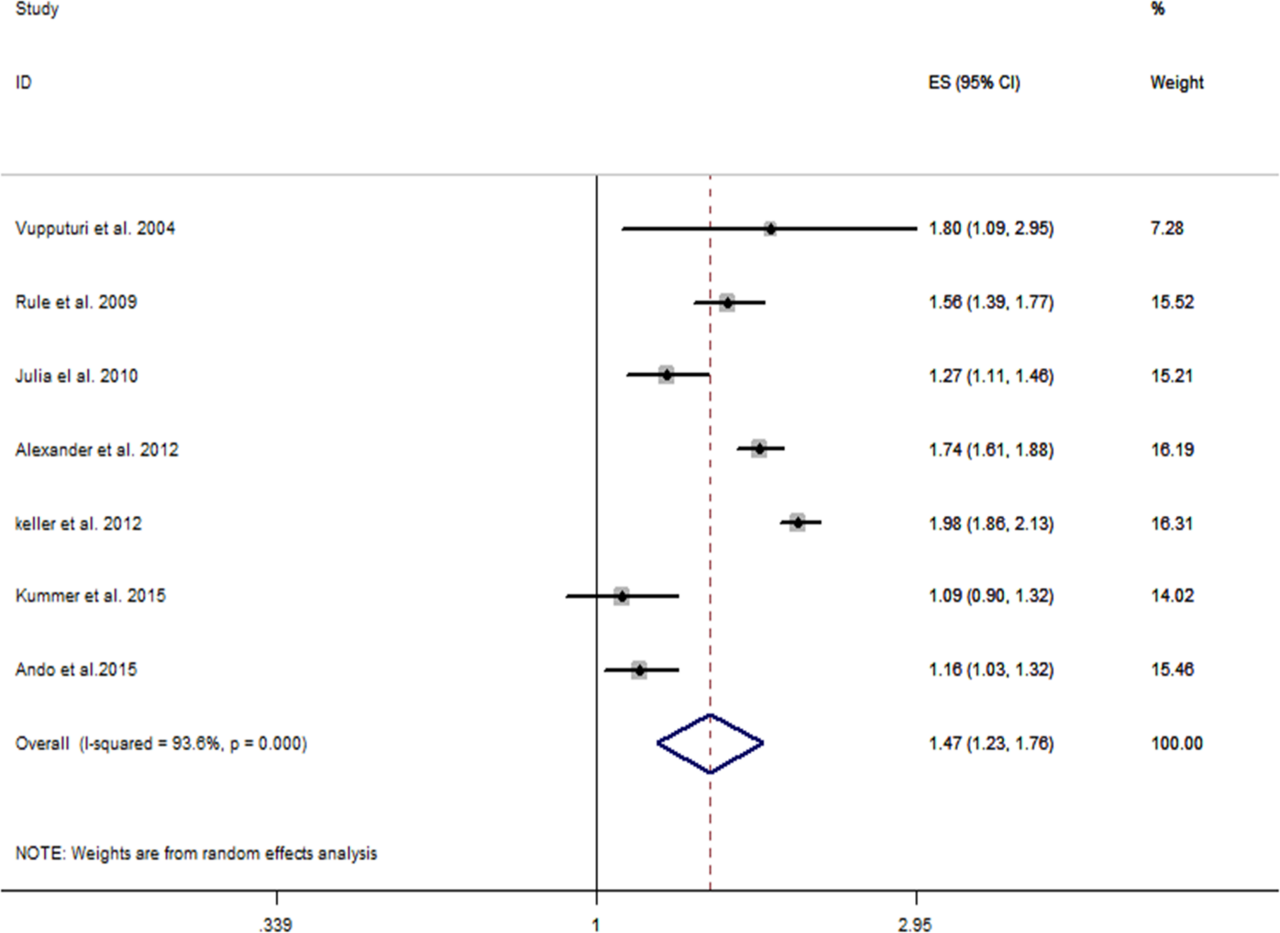
Forest plot of the included studies comparing risk of CKD between patients with a history of kidney stones and those without a history of kidney stones.

As shown in Fig. 2, the multivariate-adjusted RR of CKD within the seven individual study populations ranged between 1.09 and 1.98, with an overall multivariate-adjusted RR of 1.47 (95% CI [1.23–1.76]). Significant heterogeneity was observed (*I*^2^ = 93.6%, *P* < 0.001).

**Table 1:** Characteristics of included studies.

Study	Design	Country	Sample size	Average age (y)	Mean follow-up (y)	Diagnosis of renal stone	CKD diagnosis	Participants with baseline CKD excluded	Adjusted confounders
Vupputuri et al. (2004)	Case-control study	United States	1,062	62	NA	Telephone interviews	ICD-9 discharge diagnoses and comprehensive chart reviews	Yes	Age, race, sex, income, BMI, daily cola consumption, analgesic use, and history of hypertension, gout, multiple urinary tract infections, and pyelonephritis
Rule et al. (2009)	Cohort study	United States	14,216	44	8.6	ICD-9 codes 592, 594, and 274.11 and equivalent Hospital Adaptation of the International Classification of Diseases 8 codes	ICD-9 and equivalent HICDA-8 codes	Yes	Age, gender, hypertension, diabetes, obesity, dyslipidemia, gout, alcohol abuse, tobacco use, coronary artery disease, heart failure, cerebral infarct, and PVD
Hippisley-Cox & Coupland (2010)	Cohort study	England	775,091	47.3	5	Based on diagnosis or operative procedure at baseline	Kidney transplant; kidney dialysis; diagnosis of nephropathy; persistent proteinuria; or GFR <45 mL/min/1.73 m^2^	Yes	Age, BMI, Systolic blood pressure, Smoking status, Ethnic, Townsend score, type 1 diabetes, type 2 diabetes, cardiovascular disease, RA, Treated hypertension, congestive cardiac failure, PVD, SLE, Two or more prescriptions for NSAIDs drugs in the 6 months before study entry, Recorded family history of kidney disease including polycystic kidneys
Alexander et al. (2012)	Cohort study	Canada	1,954,836	45.9 (no stone) 51.6 (stone)	8.5	Physician claims, data on use of hospitalisation and ambulatory care, and ICD-9 codes (592, 594, 274.11) and ICD-10 codes (N20.0, N20.1, N20.2, N20.9, N21.0, N21.1, N21.8, N21.9, N22.0, N22.8)	eGFR <45 ml/min/1.73 m^2^	Yes	Age, sex, Aboriginal, receipt of social assistance, rural residence, comorbidities (Charlson score and hypertension), and eGFR
Keller, Chen & Lin (2012)	Case-control study	China	42,948	62.3	NA	ICD-9-CM codes 592, 592.0; Only selected patients who had received two or more UC diagnoses before the index date, with at least one being made by a urologist or nephrologist	ICD-9-CM code 585 (chronic renal failure) or 593.9 (unspecified disorder of kidney and ureter)	Yes	Age, patient’s monthly income, urbanisation level, geographic region, PVD, SLE, hypertension, diabetes, CHD, hyperlipidaemia, obesity, gout, anaemia, and alcohol abuse/alcohol dependence syndrome
Kummer et al. (2015)	Cohort study	United States	10,678	62.5	12	A combination of self-report and diagnostic codes	Diagnostic codes from linkage to hospitalizations and US Centers for Medicare and Medicaid Services’ records	Yes	Age, sex, race, and study center, HDL, hypertension, urine ACR, eGFR, plasma uric acid, diuretic use, smoking status, BMI, diabetes, history of CHD, and hsCRP
Ando et al. (2015)	Cohort study	Japan	11,402	NA	3.8	NA	eGFR < 60 ml/min/1.73 m^2^	Yes	Overweight/obesity, hypertension, diabetes mellitus, gout/hyperuricemia, and dyslipidemia, lifestyles

**Notes.**

NA not available CKD chronic kidney disease BMI body mass index PVD peripheral vascular disease WHR Waist/hip LDL low-density lipoprotein UC urinary calculus CHD coronary heart disease SLE systemic lupus erythematosus eGFR estimated glomerular filtration rate HDL high-density lipoprotein hsCRP high-sensitivity C-reactive protein HR hazard ratio CI confidence interval ACR albumin–creatinine ratio RA rheumatoid arthritis

### Subgroup analyses

In subgroup analyses, case-control studies showed a significantly higher CKD risk (RR 1.98; 95% CI [1.85–2.11]) in individuals with kidney stones compared with cohort studies (RR 1.35; 95% CI [1.12–1.63]). Stratification based on sample size resulted in a pooled RR of 1.46 (95% CI [1.12–1.91]) in small sample studies and 1.49 (95% CI [1.10–2.03]) in large sample studies. Stratification based on region resulted in a pooled RR of 1.52 (95% CI [0.90–2.57]) in Asian populations and 1.44 (95% CI [1.20–1.73]) in Western populations. The observed positive association was also similar among studies with a mean age <50 years (RR 1.52; 95% CI [1.27–1.82]) and those with a mean age ≥ 50 years (RR 1.56; 95% CI [0.98–2.48]), although the latter did not reach statistical significance. Stratification based on follow-up time resulted in a pooled RR of 1.21 (95% CI [1.10–1.32]) for studies with a follow-up time <7.5 years and a pooled RR of 1.46 (95% CI [1.17–1.83]) for studies with a follow-up time ≥ 7.5 years. Stratification based on gender indicated that the pooled risks for CKD remained significant in female patients with kidney stones (RR 1.57; 95% CI [1.04–2.38]). However, the risk was slightly lower and did not remain significant in male patients with kidney stones (RR 1.40; 95% CI [0.98–1.99]). High heterogeneitywas present in subgroup analyses with the exception of case-control studies and mean follow-up time <7.5 years (*I*^2^ = 0%). In addition, when meta-regression performed, none of these variables had a significant interaction with the risk estimate (all *P* values > 0.05) (Table 2).

**Table 2:** Subgroup analyses of chronic kidney disease in patients with kidney stones.

Subgroup	No. of studies	RR (95% CI)	*I*^2^ (%)	*P*^a^	*P*^b^
Study design					
Case-control	2	1.98 (1.85, 2.11)	0	0.710	0.101
Cohort	5	1.35 (1.12, 1.63)	91.5	<0.001	
Region					
Asian	2	1.52 (0.90, 2.57)	98.2	<0.001	0.808
Non-Asian	5	1.44 (1.20, 1.73)	86.7	<0.001	
Sample size					
<50,000	5	1.46 (1.12, 1.91)	94.9	<0.001	0.917
≥50,000	2	1.49 (1.10, 2.03)	93.5	<0.001	
Participant’s average age (y)					
<50	3	1.52 (1.27, 1.82)	87.2	<0.001	0.908
≥50	3	1.56 (0.98, 2.48)	94.0	<0.001	
Mean follow-up (y)					
<7.5	2	1.21 (1.10, 1.32)	0	0.337	0.380
≥7.5	3	1.46 (1.17, 1.83)	90	<0.001	
Gender					
Men	2	1.40 (0.98, 1.99)	95.3	<0.001	0.714
Women	2	1.57 (1.04, 2.38)	94.4	<0.001	

**Notes.**

RR risk ratio CI confidence interval

a *P* value for heterogeneity among studies assessed with Cochran’s *Q* test.

b *P* value for interaction evaluated by meta-regression models.

### Sensitivity analyses and reporting bias

Sensitivity analysis indicated that the estimate remained significant despite omission of any of the studies [RR varied between 1.38 (95% CI [1.16–1.65]) and 1.54 (95% CI [1.29–1.85])] (Table S2). We did not construct a funnel plot as they are known to be unreliable when used with fewer than 10 studies (Higgins & Green, 2011).

## Discussion

This study is the first meta-analysis to present the risk of CKD in patients with kidney stone history. We confirmed kidney stones were associated with an increased risk of CKD with a RR of 1.47 (95% CI [1.23–1.76]).

As with other published meta-analyses of this type (Cheungpasitporn et al., 2015; Yan et al., 2014), our study has a high level of heterogeneity. We conducted subgroup analyses and meta-regression analyses to explore sources of heterogeneity. The risk of CKD remained approximately 1.5 in all subgroups. The lack of a significant effect in Asians, those aged ≥50 years and males is notable. For example, a low incidence of kidney stones in Asians and a high prevalence of CKD in the white respondents (Jha et al., 2013; Romero, Akpinar & Assimos, 2010) may contribute to these results. However, it is also possible that the differences in information size (the non-Asian population of 2,755,883 in our analysis was far larger than the Asian population of 54,340) may explain these findings. This conclusion is supported by the results of the meta-regression, which showed that study design, region, sample size, mean age, mean follow-up time, and gender explained little of the variation between studies.

Similarly, our study indicated that female patients with kidney stones showed a slightly higher risk for CKD than male patients. The increased risk was not significant in male patients with kidney stones. It is unclear if this represents a difference in underlying pathophysiology. Differences by sex are not infrequent. In Hippisley-Cox & Coupland’s (2010) or Shoag et al.’s (2014) study, only female patients with kidney stones history showed a significant increased risk of developing moderate to severe chronic kidney disease. A similar finding has also been observed in a meta-analysis on the association of kidney stones and risk of incident coronary heart disease (Ferraro et al., 2013). The incidence of other risk factors for CKD, such as hypertension (Gillen, Coe & Worcester, 2005) and diabetes (Ando et al., 2011), also was found to be significantly higher in women but not in men with kidney stones. Further studies are needed to explore sex and racial differences in the association between kidney stones and CKD.

Note that the CKD risk in patients with kidney stones becomes more substantial when we restrict analyses to cohort studies and studies with follow-up time ≥7.5 years. The risk of CKD was lower in cohort studies versus case-control studies (RR 1.35 vs 1.98), which is important as well-conducted cohort studies provide a higher level of evidence than case-control studies. It may be that the risk of CKD associated with kidney stones is not as high as our primary analysis would suggest. Regarding follow-up time, although not a significant difference, the risk was higher in studies with ≥7.5 years follow up versus those with <7.5 years, which may reflect the slowly progressive natural history of CKD.

The pathophysiologic relation between kidney stones and new-onset CKD is rather unclear, but several potential reasons may explain the observed associations. First, for sterile stones, repeated episodes of obstruction associated with kidney stones may cause a sequence of tubular cell events followed by macrophage interstitial infiltration including an inflammatory cascade and interstitial fibroblast production, which can lead to glomerulosclerosis, reduced glomerular filtration rate, and renal insufficiency (De Water et al., 1999; Gambaro, Favaro & D’Angelo, 2001). Second, for infection-related stones, stones form in the presence of highly alkaline urine and these stones may begin to obstruct calyces and renal pelvis (Bichler et al., 2002; Viers et al., 2015), besides, ammonium ions in alkaline urine are also highly toxic and can injure or kill healthy cells (Gambaro, Favaro & D’Angelo, 2001). These factors contribute to CKD. Third, CKD is a recognized complication of kidney stones as a result of rare hereditary disorders such as primary hyperoxaluria and cystinuria (Leumann, 1985; Worcester et al., 2006). Fourth, stone treatment itself may account for the excess risk of kidney damage. Animal experiments have confirmed that extracorporeal shock wave lithotripsy disrupts the tubular basement membrane (Evan et al., 1991). Fifth, kidney stones and CKD may share responsible causal risk factors.

Several limitations of this meta-analysis should be addressed. First, significant heterogeneity existed across the studies, thus, we conducted a subgroup analysis and meta-regression analysis. Unfortunately, we could not thoroughly explain the source of heterogeneity, suggesting other unknown confounding variables may be the source of heterogeneity. Second, the meta-analysis included studies with different study designs, follow-up time, diagnostic criteria, and adjusted confounders, which could introduce inherent limitations. For example, the cut-off points used to define CKD such as eGFR <45 mL/min/1.73 m^2^ vs eGFR <60 mL/min/1.73 m^2^ differ substantially in terms of both prevalence and associated morbidity. Third, we did not have information on stone composition for most of participants with kidney stone history, and thus cannot evaluate the specific risk associated with different stone types. Similar limitations were seen on stone burden, medications, lithotripsy, other surgeries and genetic risk factors. Finally, although all the included studies controlled for several known risk factors for CKD, residual confounders may be present due to the observational nature of our study.

In conclusion, our study suggests a history of kidney stones is significantly associated with increased risk of CKD. Further efforts should be made to explore the potential biological mechanism to confirm these findings and they will stimulate the development of more effective preventive and therapeutic measures.

##  Supplemental Information

10.7717/peerj.2907/supp-1 Data S1 Raw data

10.7717/peerj.2907/supp-2 Supplemental Information 1 PRISMA checklist

10.7717/peerj.2907/supp-3 Supplemental Information 2 Supplemental Tables

## Additional Information and Declarations

### Data Availability

The following information was supplied regarding data availability: The raw data has been supplied as a Supplementary File.
