# Supplementary material for: History of kidney stones and risk of chronic kidney disease: a meta-analysis
Source: PeerJ. 2017 Jan 24;5:e2907. doi: 10.7717/peerj.2907 (PMC5267565; doi:10.7717/peerj.2907)
Supplement: Supplemental Information 2 [file peerj-05-2907-s003.doc]

**Table S1** **Assessment of study quality.**

| **References** | **Quality indications form of Newcastle-Ottawa Scale** | | | | | | | | | **Total stars** |
| --- | --- | --- | --- | --- | --- | --- | --- | --- | --- | --- |
|  | **1** | **2** | **3** | **4** | **5A** | **5B** | **6** | **7** | **8** |  |
| Cohort | | | | | | | | | | |
| Rule et al. 2009 | Yes | Yes | Yes | Yes | Yes | Yes | Yes | Yes | Yes | 9 |
| Julia et al. 2010 | Yes | Yes | Yes | Yes | Yes | Yes | Yes | Yes | No | 8 |
| Alexander et al. 2012 | Yes | Yes | Yes | Yes | Yes | Yes | Yes | Yes | Yes | 9 |
| Kummer et al. 2015 | Yes | Yes | No | Yes | Yes | Yes | Yes | Yes | No | 7 |
| Ando et al. 2015 | No | Yes | No | Yes | No | Yes | Yes | No | No | 4 |
| Case-control | | | | | | | | | | |
| Vupputuri et al. 2004 | Yes | Yes | Yes | Yes | Yes | Yes | Yes | Yes | No | 8 |
| Keller et al. 2012 | No | Yes | Yes | Yes | Yes | Yes | Yes | Yes | No | 7 |

For cohort studies: 1, exposed cohort truly or somewhat representative; 2, nonexposed cohort drawn from same community as the exposed cohort; 3, ascertainment of exposure; 4, outcome of interest not present at start; 5A, cohorts comparable on basis of age; 5B, cohorts comparable on any additional factor; 6, assessment of outcome (independent blind assessment or record linkage); 7, follow-up ≥5 y; 8, complete accounting for cohorts or subjects lost to follow-up unlikely to introduce bias; For case–control studies: 1, cases independent validation; 2, cases are consecutive or representative; 3, community controls; 4, controls have no history of endpoint; 5A, study controls for age; 5B, study controls for any additional factor; 6, assessment of exposure (independent blind assessment or record linkage); 7, same method of ascertainment used for cases and controls; 8, same non-response rate for both groups.

**Table S2 Sensitivity analysis.**

| Study omitted | RR | 95%CI | | I^2^ (%) | *P*^a^ |
| --- | --- | --- | --- | --- | --- |
| Vupputuri et al. 2004 | 1.45 | 1.20 | 1.74 | 94.6 | <0.001 |
| Rule et al. 2009 | 1.45 | 1.18 | 1.80 | 94.6 | <0.001 |
| Julia el al. 2010 | 1.51 | 1.24 | 1.83 | 93.6 | <0.001 |
| Alexander et al. 2012 | 1.42 | 1.13 | 1.80 | 94.5 | <0.001 |
| keller et al. 2012 | 1.38 | 1.16 | 1.65 | 89.5 | <0.001 |
| Kummer et al. 2015 | 1.54 | 1.29 | 1.85 | 93.3 | <0.001 |
| Ando et al.2015 | 1.53 | 1.29 | 1.82 | 91.6 | <0.001 |

RR, risk ratio; CI, confidence interval.

^a^ *P* value for heterogeneity among studies assessed with Cochran’s Q test.
